# Pan-neutralizing, germline-encoded antibodies against SARS-CoV-2: Addressing the long-term problem of escape variants

**DOI:** 10.3389/fimmu.2022.1032574

**Published:** 2022-10-28

**Authors:** Justin Mark Lunderberg, Sanjucta Dutta, Ai-Ris Y. Collier, Jeng-Shin Lee, Yen-Ming Hsu, Qiao Wang, Weina Zheng, Shushun Hao, Haohai Zhang, Lili Feng, Simon C. Robson, Wenda Gao, Stefan Riedel

**Affiliations:** ^1^ Center for Inflammation Research, Department of Anesthesia, Critical Care and Pain Medicine, Beth Israel Deaconess Medical Center, Harvard Medical School, Boston, MA, United States; ^2^ Department of Pathology, Beth Israel Deaconess Medical Center, Harvard Medical School, Boston, MA, United States; ^3^ Center for Virology and Vaccine Research, Beth Israel Deaconess Medical Center, Harvard Medical School, Boston, MA, United States; ^4^ AB Biosciences, Inc., Concord, MA, United States; ^5^ Shijiazhuang Hipro Biotechnology Co., Ltd., Hebei, China; ^6^ Department of Hematology, Shandong Provincial Hospital Affiliated to Shandong First Medical University, Jinan, China; ^7^ Antagen Diagnostics, Inc., Canton, MA, United States; ^8^ Antagen Pharmaceuticals, Inc., Canton, MA, United States

**Keywords:** SARS-CoV-2, neutralizing antibodies, somatic hypermutation, B cell memory, vaccines, IgM, B-1 B cells, Reverse Vaccinology

## Abstract

Despite the initially reported high efficacy of vaccines directed against ancestral SARS-CoV-2, repeated infections in both unvaccinated and vaccinated populations remain a major global health challenge. Because of mutation-mediated immune escape by variants-of-concern (VOC), approved neutralizing antibodies (neutAbs) effective against the original strains have been rendered non-protective. Identification and characterization of mutation-independent pan-neutralizing antibody responses are therefore essential for controlling the pandemic. Here, we characterize and discuss the origins of SARS-CoV-2 neutAbs, arising from either natural infection or following vaccination. In our study, neutAbs in COVID-19 patients were detected using the combination of two lateral flow immunoassay (LFIA) tests, corroborated by plaque reduction neutralization testing (PRNT). A point-of-care neutAb LFIA, NeutraXpress™, was validated using serum samples from historical pre-COVID-19 negative controls, patients infected with other respiratory pathogens, and PCR-confirmed COVID-19 patients. Surprisingly, potent neutAb activity was mainly noted in patients generating both IgM and IgG against the Spike receptor-binding domain (RBD), in contrast to samples possessing anti-RBD IgG alone. We propose that low-affinity, high-avidity, germline-encoded natural IgM and subsequent generation of class-switched IgG may have an underappreciated role in cross-protection, potentially offsetting immune escape by SARS-CoV-2 variants. We suggest Reverse Vaccinology 3.0 to further exploit this innate-like defense mechanism. Our proposition has potential implications for immunogen design, and provides strategies to elicit pan-neutAbs from natural B1-like cells. Refinements in future immunization protocols might further boost long-term cross-protection, even at the mucosal level, against clinical manifestations of COVID-19.

## Introduction

As of mid-2022, more than 560 million people globally have been infected with severe acute respiratory syndrome coronavirus 2 (SARS-CoV-2), the causative agent for coronavirus disease 2019 (COVID-19). Over 6.3 million people have died of infection-mediated complications. The extraordinarily rapid development of several vaccines in the first year of field deployment has saved close to 20 million lives that would otherwise have been lost to COVID-19 (1). However, as waves of variants-of-concern (VOC) emerge, breakthrough infections by the variants in fully vaccinated individuals have become increasingly common (2–7), and may still cause substantial morbidity and mortality. Particularly, SARS-CoV-2 Omicron strains have accumulated unprecedented numbers of mutations in the Spike protein with ∼40 residue changes versus 10 on average in all the previous dominant variants (8, 9) that evade neutralizing antibody (neutAb) binding. As a result, individuals who received two doses of the BNT162b2 mRNA vaccine have over 22-fold decreases in neutralizing activity against the Omicron strain, when compared to the ancestral Wuhan-Hu-1 strain (10). Efficacies of the other current vaccines against Delta and Omicron VOCs have also declined (11).

Likewise, passive transfer of therapeutic neutralizing monoclonal antibodies (mAb) was initially successful in treating COVID-19 before the arrival of the variants (12), but many neutAbs previously approved for emergency use by the FDA do not retain efficacy against Delta and Omicron strains (13, 14). In separate studies, the neutralizing activity of the majority of tested SARS-CoV-2 mAbs were either abolished or impaired against Omicron (15, 16).

Therefore, for neutAb developers, active questions are: 1) Whether it is possible to identify, characterize and isolate pan-neutAbs against the majority of current and future SARS-CoV-2 variants and 2) if this venture is successful, whether developing recombinant pan-neutAbs as prophylactic and therapeutic modalities can stay ahead of evolving variants? These questions also apply to vaccine development to stimulate durable pan-neutralizing antibody responses.

The answer to the first question is in the affirmative. By adopting high throughput single cell sequencing of thousands of Spike-enriched memory B cells (MBCs) from the PBMCs of convalescent patients (17), researchers have identified IgG1 type pan-neutAbs, for example, DXP-604 (16) and 76E1 (18). Like VIR-7831 (Sotrovimab), DXP-604 effectively neutralizes SARS-CoV-2 D614G, Alpha, Beta, Gamma, Delta as well as Omicron (16). Unlike DXP-604, 76E1 binds to a unique S2 epitope partially buried in the pre-fusion state of the Spike trimer, which is only exposed when the Spike protein binds to ACE2. As a result, while all the other RBD-binding neutAbs bind to the pre-fusion state of Spike trimer, 76E1 does not.

Nevertheless, as the S2 regions are conserved among multiple human coronaviruses, blocking the interaction of the highly conserved S2’ site and the fusion peptide by 76E1 can effectively block virus-cell fusion and broadly neutralize seven human coronaviruses, including two α-coronaviruses (HCoV-229E and HCoV-NL63) and five β-coronaviruses (SARS-CoV-2 and all its VOCs, SARS-CoV, MERS-CoV, HCoV-OC43 and HCoV-HKU1) (18). Thus, although very rare, pan-neutAbs with unique mechanisms of action can be isolated with extensive screening efforts. These studies indicate that MBCs exhibit repertoires with various specificities to Spike glycoprotein that are enhanced by somatic hypermutation and could avoid the SARS-CoV-2 immune escape.

The answer to the second question is more complex. Large-scale manufacturing, clinical trials and regulatory approval of the recombinant neutAbs typically take a long time and are hugely expensive. In light of certain neutAbs being “highly unlikely to be active against the Omicron variant, which is circulating at a very high frequency throughout the United States”, on January 24^th^, 2022, the FDA restricted the emergency use of Bamlanivimab and Etesevimab (co-administered) and REGEN-COV (Casirivimab and Imdevimab) to “patients that are likely to have been infected with or exposed to a variant that is susceptible to these treatments”.

To avoid such restrictions in future therapeutic mAbs, researchers need to reassess if there are fundamental immunological elements missing in our current understanding of such pan-neutAbs and of the durability of vaccine-induced protection. The following sections are devoted to such mechanistic discussions, albeit with a focus on mRNA vaccination.

## NeutAbs in natural infection have greater breadth than after vaccination

Using single B cell sequencing, ELISA, biolayer interferometry and pseudovirus neutralization assays, Cho et al. compared cloned antibodies from MBCs at 1.3 and 6.2 months after natural infection in a cohort of convalescent patients, with similarly cloned antibodies from MBCs at 1.3 and 5 months after the 2^nd^ dose of either Moderna (mRNA-1273) or Pfizer-BioNTech (BNT162b2) mRNA vaccines in subjects having no prior history of SARS-CoV-2 infection (19). They found that at 1.3 and 5 months after the 2^nd^ vaccine dose, mRNA vaccines induced 4.9- and 3.6-fold higher plasma neutAb titers than natural infection at 1.3 and 6.2 months following infection. Between the 1^st^ and 2^nd^ doses, MBCs continue to evolve antibodies with increased neutralizing activity, yet there is no further increase in potency or breadth thereafter. In contrast, individual MBC-derived antibodies selected over time by natural infection have greater potency and breadth than antibodies elicited by vaccination (19). Here, the key element is the breadth of MBCs, which directly determines the effectiveness of their secreted neutAbs against rising variants (20).

Antibody-secreting plasma cells and peripheral MBCs are independently regulated cell populations, playing different roles in the maintenance of protective humoral immunity. The initial burst of short-lived plasmablasts (21, 22) produces sufficient concentrations of neutAbs in the circulation to protect an individual at high risk of exposure to SARS-CoV-2. However, populations of plasmablasts and circulating plasma cells contract quickly and, as a result, the serum neutAb titers wane significantly over a period of months following antigen stimulation. Our own work with an LFIA test NeutraXpress™ has further confirmed this rapid waning of mRNA vaccine-induced neutAb titers, at 3-6 months after the 2^nd^ dose (23). The kinetics of decreasing neutAb titers correlates with reports of reinfection in convalescent individuals and breakthrough infection by variants in fully vaccinated individuals (7, 24).

On the other hand, MBCs are responsible for swift recall responses to previously experienced epitopes. The number of quiescent MBCs is relatively stable over the first 5–6 months after mRNA vaccination or natural infection (25); during this period the cells undergo somatic hypermutation for increased antibody affinity (19, 26). At the molecular level, while 19% and 21% of the cloned antibodies recovered from single MBCs of vaccinated individuals have shown improved potency and greater breadth, respectively, these numbers for similarly cloned antibodies from convalescent patients are 59% and 69% (19). At the population level, SARS-CoV-2-naïve individuals who received two doses of the BNT162b2 mRNA vaccine exhibited a 13.06-fold increased risk for breakthrough infection with the Delta variant, when compared with previously-infected individuals who have not been vaccinated (24). Thus, natural infection-elicited neutAbs from MBCs show greater neutralizing potency and breadth than those induced by vaccination over a similar period of time (19, 24).

As broad antigen recognition is crucial for potential induction of pan-neutAbs to prevent both infection and disease caused by VOCs, it is important to understand the immunological processes that shape the breadth of neutAbs.

## Low-affinity cross-reactive MBCs provide rapid and potent recall responses towards antigenic variants

Primary germinal center (GC) responses drive the development of two distinct but equally important B cell populations: the high-affinity long-lived plasma cells (LLPCs) (27, 28) generated by activation-induced cytidine deaminase (AID)-driven somatic hypermutation and the diverse pool of largely AID-independent rarely-mutated MBCs. LLPCs provide protective immunity by producing high-affinity circulating antibodies against the same re-encountered (homologous) pathogen, whereas MBCs induce a rapid, first-line antibody response to infections by secreting a diverse pool of cross-reactive antibodies that can recognize classes of related (heterologous) pathogens or recognize rapidly and continuously mutating pathogen variants (29, 30).

For example, upon infection or immunization with one flavivirus (West Nile virus, WNV), the low-affinity cross-reactive MBCs generated had a very limited capacity to re-enter secondary GCs, and were excluded from GC-somatic hypermutation. However, these MBCs developed enhanced affinities towards a heterologous flavivirus (Japanese Encephalitis virus, JEV) by forming extrafollicular plasmablasts (31). Lineage tracing and antibody cloning from single B cells also showed that a large fraction of the GC B cells express high-affinity B cell receptors, whereas the vast majority of simultaneously selected MBCs express receptors with very low-affinity for the antigen (32). In B cell receptor (BCR) transgenic mice, more MBCs were produced when mice were challenged with lower affinity antigens (33). It was also found that nascent MBCs require high valency interactions between their BCR and multimerized antigen; this increases the apparent affinity through avidity effects, resulting in a MBC population with much lower overall affinities than the contemporaneous GC B cells (32), the latter of which are the major source for the bone marrow (BM)-residing LLPCs.

Taken together, these studies address a critical question of how MBCs respond to heterologous challenges, *i.e.*, whether responses are through creating fundamentally new specificities through secondary GCs or through the selection of pre-existing clones without further affinity maturation. Unlike antigen-specific LLPCs derived from high-affinity naïve B cells, precursors of MBCs encoding low-affinity antibodies with germline sequences largely bypass secondary GCs in recall responses (34). Similarly, recall of MBCs with germline sequences, rather than activation of naïve, mature B cells, has been the primary component of the response to influenza strains exhibiting antigenic drift (35). Thus, the recall responses are restricted by clonal selection from the panel of pre-existing MBC specificities with limited contributions (25) from further affinity maturation (29, 30). Therefore, such low-affinity cross-reactive MBCs may contribute to the protection against future variants.

## Early neutAbs against SARS-CoV-2 are enriched with germline sequences, and may have an IgM+ innate-like B-1 cell origin

In a relatively short longitudinal analysis (8-69 days after diagnosis) of SARS-CoV-2-infected subjects, Kreer and colleagues found highly potent neutAbs developed early after infection and exhibited limited ongoing somatic hypermutation (36). From sorted single B cells, 31 of 79 binding and 10 of 27 neutralizing antibodies exhibited 99%–100% germline identities, with no correlation between neutralizing activity and the level of somatic mutation. The potential precursor sequences of the SARS-CoV-2 binding and neutralizing antibodies were even identified as near-germline sequences (preference for IGHV3-30) in naïve B cell repertoires from healthy individuals, before the COVID-19 pandemic (36). The usage of near-germline sequences (*e.g.*, IGHV3-53 and IGHV3-66) by SARS-CoV-2 specific antibodies in the early response has also been confirmed by other studies (34, 37–45). Some potent therapeutic neutAbs are found to utilize germline sequences as well, including one which obtained emergency use authorization (CB6/LY-CoV016) (40) and several currently under clinical investigation (P2C-1F11/BRII-196 and BD-604/DXP-604) (46, 47). These studies suggest that neutAbs can be readily generated from existing germline antibody sequences found in the general population (48), a feature reminiscent of natural IgM-expressing B-1 cells in mice.

The B cell compartment can be divided into two developmentally and functionally distinct populations, B-1 and B-2 B cells (49). B-1 cells are primarily derived from the fetal liver, whereas the conventional B-2 cells originate from the BM and can be further characterized into follicular B (FOB) and marginal zone B (MZB) cells (50, 51). B-1 cells are found in the body cavities and in mucosal tissues as well, including the lamina propria of the gut and the respiratory tract. During respiratory infections, B-1 cells in the pleural cavity (accounting for 35% - 70% of total B cells found in this site) can produce large amounts of IgM and IgA natural antibodies as a first-line defense against pathogens (52). Innate natural antibodies are primarily encoded by germline sequences with minimal N-region addition and without somatic hypermutation; they are functionally important for early pathogen clearance (53). Recently, an elusive human B-1 cell population, equivalent to the well-studied mouse counterpart, has been proposed to have the unique surface phenotype CD20+CD27+CD43+, distinguishing it from other B cell subsets (54, 55). While controversy on precise phenotyping is still present, it is anticipated that, when compared to murine B-1 cells, the human analogue will possess a similar lineage and function.

It is important to understand the origin of neutAbs for SARS-CoV-2. B-1 cell antibodies are selected for function (*e.g.*, defense against microbial pathogens in innate immunity), and B-2 cell antibodies are selected for affinity to a pathogen (55). NeutAbs of B-1 ontogeny may initially have lower affinity and elevated cross-reactivity due to their origin of germline natural IgM (56). Indeed, of the 27 early-stage SARS-CoV-2 neutAbs isolated by Kreer et al., 4 showed low-to-moderate levels of autoreactivity and 2 showed cross-reactivity towards heterologous envelope proteins (Ebola glycoprotein and HIV-1 gp140) (36). This feature may allow such B cells to be preferentially recruited to the extrafollicular MBC compartment and subsequently evolve their neutAbs (*via* class-switching and low-degree somatic hypermutation) into antibodies with greater breadth and affinity to rising variants. To the contrary, because of the BCR sequence use, the malleability of neutAbs of B-2 ontogeny may be limited and these might be funneled towards high affinity with a LLPC host cell fate, targeting only the original strain. These neutAbs are highly specific to the homologous immunogen and yet are not flexible in their response and are eventually outpaced by a rapidly-mutating pathogen.

To test this hypothesis, we proposed these studies: 1) Whether neutAb activities can be detected in the early phase of SARS-CoV-2 infection (<40 days post onset of symptoms); and 2) Whether RBD-specific IgM contributes to the serum neutralizing activity. First, we stratified PCR-confirmed COVID-19 patients based on days following symptom onset and IgM/IgG profiles of anti-RBD antibodies in their serum samples. For that, we used an LFIA rapid test DISCOVID™ to detect the presence of RBD-specific IgM and/or IgG in their preserved serum samples. The specificity (96.6%) and sensitivity (92.7%) of DISCOVID™ have been previously determined with historical samples collected prior to the pandemic and in PCR-confirmed COVID-19 patients (**Supplementary Tables 1** and **2**). Therefore, patients were stratified into 4 subgroups of RBD reactivity: IgM+IgG-, IgM+IgG+, IgM-IgG+, and IgM-IgG-. Then, we used the second LFIA rapid test NeutraXpress™ to detect the presence of neutAbs in each subgroup, and to correlate with the time following symptom onset or PCR positivity in asymptomatic patients. The rapid test NeutraXpress™ with a double-lane design for simultaneous side-by-side comparison of neutAb activity in patient samples with buffer control has been described in field testing of healthy individuals following mRNA vaccination (23). NeutraXpress™ was also validated with PRNT_90_ assay of PCR-confirmed COVID-19 samples with a high sensitivity (88%) to detect PRNT_90_ ≥ 80 neutAb activity (**Supplementary Figures 1** and **2**, **Supplementary Tables 3** and **4**).

**Figure 1:**
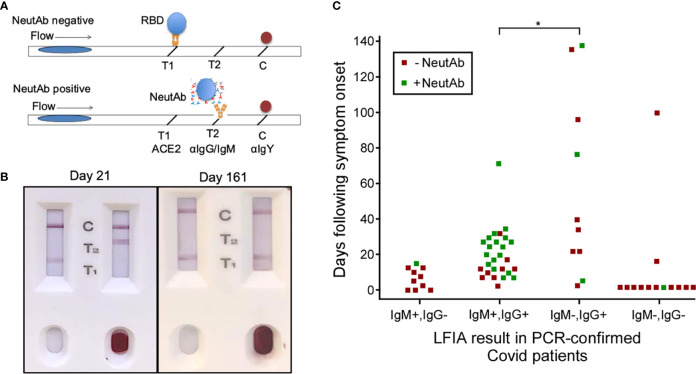
NeutAbs against SARS-CoV-2 detected with NeutraXpress™ in the early phase of natural infection are enriched in COVID-19 patients possessing the IgM+ signature. **(A)** Illustration of NeutraXpress™ design (23). T1 is striped with recombinant His-tagged human ACE2 protein. T2 is striped with anti-human IgM + IgG Abs. The conjugate pad is impregnated with colloidal gold nanoparticles (GNP)-labeled recombinant RBD from Spike protein of SARS-CoV-2, as well as GNP-labeled chicken IgY used as a tracer to indicate the completion of the lateral flow when it is captured by goat anti-chicken antibody at the C line. If there is no neutAb or binding antibody in the specimen, GNP-RBD is captured by ACE2 at T1 line and the T2 line should not appear. If the specimen contains neutAbs, the interaction between GNP-RBD with ACE2 at the T1 line is blocked and T1 disappears or shows reduced intensity, in comparison with T1 from the control well with added diluent only. The appearance of the T2 line indicates the presence of IgM and/or IgG Abs specific for RBD, *i.e.*, T2 shows the totality of both neutralizing and non-neutralizing RBD-binding IgM and IgG Abs. T2 intensity correlates with higher titers for RBD-binding IgM + IgG Abs, but T2 does not provide information on neutAbs. **(B)** Examples of NeutraXpress™ showing blood samples from healthy subjects at 21 days (left) and 161 days (right) after receiving the 2^nd^ dose of mRNA vaccine. Note that the left sample wells were added with diluent only, whereas the right sample wells were added with 1 drop of whole blood. **(C)** PCR-confirmed COVID-19 patients were sub grouped based on their serum IgM/IgG profiles of anti-RBD reactivity, using an LFIA rapid test DISCOVID™ and graphed according to the number of days following symptom onset or PCR positivity (in asymptomatic patients) that the sample was taken. Each square symbol represents one patient. Each data point was further differentiated to show the presence of neutAbs in the serum samples as determined by a second LFIA rapid test NeutraXpress™. Green coloration indicates the presence of neutAbs and red coloration indicates the absence of neutAbs. Samples positive for SARS-CoV-2 neutAbs were concentrated in patients having an IgM+IgG+ anti-RBD profile, compared with patients that were anti-RBD IgG+ only (*P <0.05, two-sided two proportion z test).

Intriguingly, samples in IgM-IgG+ subgroup fulfill two requirements for neutAb generation: 1) adequate time post symptom onset; and 2) IgG isotype RBD reactivity, indicative of post-GC events. However, in direct comparison to IgM+IgG+ subgroup (66.7% samples neutAb positive), there appears to be much lower neutralization activity in IgM-IgG+ subgroup (30% samples positive) (*P*<0.05) (**Figure 1**). Our observation that neutAbs maybe encoded by innate IgM during early SARS-CoV-2 infection (detectable in patients with symptom onset time of less than 40 days) is in line with a previous report demonstrating that neutAbs isolated on days 8–17 and days 34–42 after COVID-19 diagnosis showed 97.2% and 97.0% VH gene germline identities, respectively (36). Such innate IgM-expressing B cells, possibly B-1 cells enriched at the infection site (pleural cavity), might be preferentially engaged in the development of SARS-CoV-2 neutAbs.

## Different B cell subsets are engaged by particulate antigen *vs*. soluble antigen

There are three distinct features that may precipitate the different immunologic outcomes of natural infection and mRNA vaccination: 1) route of antigen exposure (respiratory track *vs*. intramuscular inoculation); 2) antigen dose and persistence (weeks *vs*. days); and 3) antigen form [particulate intact virus *vs*. cell-surface displayed Spike trimer locked in a stabilized pre-fusion state (57)]. Notably, antigens in particulate form and soluble form can trigger distinct immunological responses (58). Upon injection of an mRNA vaccine, leukocytes are attracted to the injection site and take up the vaccine formulation. The mRNA-mediated *de novo* expression of the vaccine antigen is similar to the presentation of engulfed soluble antigens by antigen-presenting cells (APCs); following vaccination, full length Spike protein expressed by monocytes, macrophages and dendritic cells (DCs) stimulates conventional B-2 cells in the axillary lymph nodes *via* the classical MHC class I and class II antigen presentation pathways (57). In support of this notion, it has been reported that soluble Spike protein can be detected in the plasma of 96% of subjects 1-2 days following receipt of their first mRNA vaccine (median Spike concentration of 47 pg/mL) and in 63% of subjects 7 days post vaccination (median Spike concentration of 1.7 pg/mL) (59). Nevertheless, when encountering repetitive antigens on intact viruses or on particulate virus-like particles (VLPs), antigen-specific B cells but not DCs are the dominant APCs, and are sufficient to stimulate T follicular helper (Tfh) cells (60). While DCs are required to present soluble antigens for CD4+ T cell development, DCs are dispensable but B cells are engaged for the initial CD4+ T cell activation when particulate antigens are presented (60). Thus, human B-1-like cells with low-affinity and potentially cross-reactive BCR enriched in the pleural cavity and lung mucosa are likely to be differentially activated by the high valency Spike proteins displayed on virus particles during natural infection. These B-1-like cells meet the requirements for affinity restriction and have the repertoire diversity to develop into MBCs, and are likely the endogenous resource for pan-neutAbs against future mutated pathogen variants.

## Perspective on the design of broadly protective vaccines for COVID-19

Widespread danger from SARS-CoV-2 still lingers, due to the evolution of new escape variants impervious to current vaccination strategies. A broader sobering outlook includes the estimation that 58% (218 out of 375) of infectious diseases currently confronted by humanity worldwide may be aggravated by the effects of climate change in the near future (61). There is a long and ever-growing list of desired vaccines, and yet fewer than 30 pathogens have vaccines licensed for human use (www.who.int/immunization/diseases/en). For pathogens with strong immune-evasion potential, what could be the ideal strategy to aid the development of pan-protective vaccines against the circulating strains and emerging variants?

### Reverse vaccinology 3.0

More than half of the currently licensed vaccines were developed with the classical vaccinology tenants of three “I”s: Isolate, Inactivate and Inject (62). In recent years, more advanced technologies in sequencing pathogen genomes and single B cells, aided with highly sophisticated means for protein structural analysis, have been used in identifying vaccine candidates by “Reverse Vaccinology”. In Reverse Vaccinology 1.0, vaccine antigens are selected *in silico* using the genomic sequence information of the pathogen without the need for growing the specific microorganism. This strategy directly led to the successful development of the meningococcal B vaccine (63).

In Reverse Vaccinology 2.0, neutAbs are used to identify protective antigens/epitopes, and to derive structural information to guide the immunogen design (64, 65). For example, the metastability of the surface F glycoprotein of respiratory syncytial virus (RSV) causes its pre-fusion form to readily decay to the post-fusion form. Antibodies against the post-fusion form bind poorly to the pre-fusion form and do not neutralize the virus effectively. When the quaternary epitope at the apex of F trimer was revealed to be bound by a pre-fusion locking neutAb, disulfide linkages and cavity-filling mutations were introduced to generate a stabilized F protein as an immunogen, which induced excellent RSV-neutralizing titers in animal models and showed great promise as a potential human vaccine (66, 67). A similar strategy of studying the structural interactions between neutAbs and immunogen for more rational design has been adopted to derive the uncleaved prefusion-optimized (UFO) envelope protein of HIV-1 as a highly promising vaccine candidate for HIV (68).

**Figure 2:**
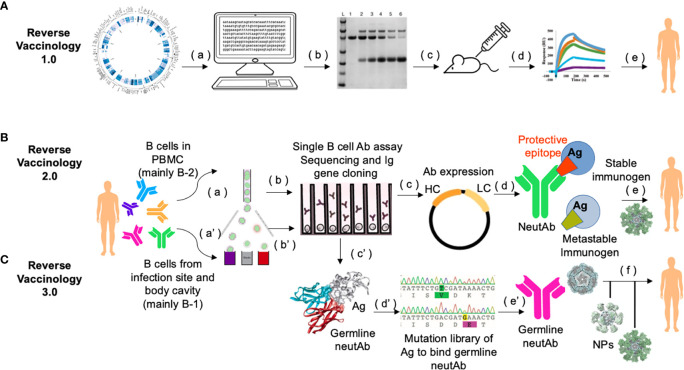
Workflow of Reverse Vaccinology 3.0. **(A)** In Reverse Vaccinology 1.0, complete genome sequences of pathogens are analyzed and genes coding for surface exposed proteins are identified (a). Potential surface-exposed proteins are expressed (b), and used to immunize the mice (c). Immunogens that can most efficiently elicit protective responses are screened (d), and further optimized as clinical trial leads for human vaccines (e). This process obviates the need to directly culture the pathogen. **(B)** In Reverse Vaccinology 2.0, structural data are utilized to guide immunogen design. First, MBCs or plasmablasts from PBMCs of subjects seropositive through infection are enriched and sorted (a). Then sorted single B cells are cultured and stimulated to screen for antigen-specific B cell clones in neutralization assays and their paired VH and VL genes are PCR amplified and sequenced (b). Recombinant neutAbs are expressed in mammalian cells, *e.g.*, HEK293 cells or CHO cells (c), to obtain sufficient materials for function confirmation in animal models (not shown), and for structural characterization of such neutAbs bound to their target antigen (Ag). Co-crystallization analysis of neutAb (usually Fab) and antigen provides detailed structural information of the protective epitope or the conformation of the antigen in general, which is different from the one in the metastable form of the antigen that often can fail to induce neutAbs (d). Protein engineering is guided by 3D modeling to stabilize the monomer immunogen and present it in a multimeric nanoparticle format as a potential candidate for human vaccine (e). **(C)** In Reverse Vaccinology 3.0, the goal is to design a germline-antibody-binding immunogen and gradually evolve germline neutAbs into ones with sufficient breadth to neutralize the current pathogen and its future variants. This process starts by enriching of B cells from the first-line of defense, *i.e.*, fluids in the body cavity and mucosa [*e.g.*, from sputum, ref (71)], where natural antibody-secreting B-1 B cells are predominantly present (a’). Antigen-binding single B cells are functionally screened and their Ig sequences are scrutinized with software [*e.g.*, IgSCUEAL, ref (72)] to identify the germline sequences (b’). Next generation sequencing of enriched B-1 B cells from a cohort of infected patients at early stages of the disease (*e.g.*, stratified by our method in **Figure 1**), may help identify convergent germline sequences induced by infection (38). Modeling with software such as Rosetta allows for the calculation of the interacting area between the Fab of germline neutAbs and the antigen (c’). Since the native antigen usually does not bind the germline antibody sequences (73, 74), it is necessary to generate a yeast surface displayed random mutational library of the antigen to select for variants that bind germline neutAbs as the initial immunogen (d’). Such immunogens would be presented in multimeric form on self-assembled nanoparticles during primary vaccination (e’), followed by sequential boosting with homologous and/or heterologous immunogens to facilitate somatic hypermutation and nurture broad neutAbs that also protect against future variants (f). This strategy relies heavily on computational bioinformatics, and has a species restriction on Ig repertoire, hence humanized mice with knock in human Ig locus would be the preferred preclinical animal model (75).

Based on the aforementioned discussion and work by others on neutAb sequence usage and B cell ontogeny, we propose that the era of Reverse Vaccinology 3.0 is being ushered in with the inclusion of B cell ontogeny as a major consideration (69, 70) (**Figure 2**). In this strategy, rational immunogen design for broad neutAbs relies on not only the tertiary/quaternary structural information of the pathogen antigen that is relevant to the induction of neutAbs, but also on the preferential usage of germline sequences in the elicited neutAbs. To achieve the preferential usage of germline sequences, immunogen should be displayed in a multimeric form, such as on liposomes (76, 77), synthetic protein nanoparticles (78–80), or VLPs (81) to trigger the low-affinity high-avidity germline BCRs from B-1 cells. Once germline antibodies are activated, further immunogens modeled on related variants and presented on multimeric nanoparticles could then be applied in sequential or cocktail immunizations to shepherd the antibody response towards greater breadth.

### Implications for COVID-19 vaccine enhancement

Breakthrough infections are indicative of vaccine failure. Intramuscular injections generate systemic immunity but little or no mucosal immunity in the respiratory tract, where SARS-CoV-2 enters the body. Hence, intramuscular injections are unlikely, on their own, to completely stop viral transmission, abolish community spread, and prevent the emergence of new variants. Repeated boosting with the same mRNA-based vaccines, not only isn’t the solution for this conundrum, but also could generate more complications by restricting the repertoire against SARS-CoV-2, particularly when considering the natural immune repertoire of the young. It has been demonstrated, *e.g.*, in the HIV vaccine field, that repeated immunization with the same immunogen is not effective in inducing broad neutAbs (82). For a pathogen that is prone to immune-evasion, like SARS-CoV-2, the most effective vaccination strategies should be aimed for breadth over depth.

As breadth is intrinsically associated with activating the germline-bearing natural antibodies of the B-1 B cell origin, the most relevant immunization route for COVID-19 should be to target local B-1 cells within the pleural cavity and lung mucosa, rather than using intramuscular inoculation that primarily targets B-2 B cells. For this to be successful, immunogen would need to be presented in a high valency multimeric form, *e.g*., Spike protein displayed on nanoparticles, which can stimulate potent neutAbs at a faster rate with minute doses (78). To reduce the possibility of inducing non-protective antibodies, RBD alone or in conjunction with the 76E1 epitope (18) can be displayed. In addition, including certain T cell epitopes as a peptide linker of the immunogen may also be necessary for the development of long-term T cell immunity to COVID-19 disease (83, 84). Moreover, immunogen delivery would likely need to be through a more physiologically relevant route, *i.e.*, nasal spraying or bronchial inhalation. More than a dozen nasal sprays or drops are actively being tested against COVID-19 in humans, either as a primary immunization or as a booster (85). Among them, some utilize HBV VLPs or attenuated RSV/Adenovirus (86) to display the Spike protein of SARS-CoV-2. Notably, an inhaled Adenoviral COVID-19 booster can effectively induce high titers of neutAbs against Omicron BA.5, and IgA in blood (most likely in respiratory mucosa as well) (86). Strategies which present a multimeric immunogen to harness B-1 germline antibody sequences are the first steps towards nurturing a broadly protective vaccine that not only prevents severe disease, but also may block symptomatic infection on exposure and thwart future variants if they arise.

## Conclusions

Mammalian hosts can never fully ‘‘outrun’’ pathogens, given the pathogen’s replication speed and mutation rates. Thus, chasing recombinant pan-neutralizing antibodies as prophylactic and therapeutic modalities is, at most, a temporary fix to the long-term problem of escape variants. Strategies laid out by Reverse Vaccinology 3.0 exploit the diversity and plasticity of the natural B cell repertoire pre-existing within our own body, and may help the host change the rules in the arms race between host and pathogen (30).

## Data availability statement

The original contributions presented in the study are included in the article/**Supplementary Material**. Further inquiries can be directed to the corresponding authors.

## Ethics statement

The studies involving discarded clinical samples were reviewed and approved by an IRB of Beth Israel Deaconess Medical Center, Harvard Medical School.

## Author contributions

JL, SD, A-RC, J-SL, Y-MH, HZ and LF collected data in testing the clinical samples with DISCOVID™ and NeutraXpress™, QW, WZ and SH optimized and produced the LFIA tests, WG, SCR and SR designed research conceptually and secured funding. JL, SCR and WG wrote and edited the manuscript. All authors contributed to the article and approved the submitted version.

## Funding

This research was funded by Massachusetts Life Science Center’s Accelerating Coronavirus Testing Solutions (ACTS), and SBIR 75N93019C00014, 75N93020C00042, and 75N93022C00011.

## Acknowledgment

We thank Dr. Xuemei Zhong for the insightful discussion on the basic immunological mechanisms involved in this topic.

## Conflict of interest

WG is employed by Antagen Diagnostics, Inc., which is the developer of DISCOVID™ and NeutraXpress™. WG is also employed by company Antagen Pharmaceuticals, Inc. SCR is a scientific founder of Purinomia Biotech Inc and consults for eGenesis, AbbVie and SynLogic Inc; his interests are reviewed and managed by HMFP at Beth Israel Deaconess Medical Center in accordance with the conflict-of-interest policies. Authors J-SL and Y-MH are employed by AB Biosciences, Inc. Authors WZ and SH are employed by Shijiazhuang Hipro Biotechnology Co.

The remaining authors declare that the research was conducted in the absence of any commercial or financial relationships that could be construed as a potential conflict of interest.

## Publisher’s note

All claims expressed in this article are solely those of the authors and do not necessarily represent those of their affiliated organizations, or those of the publisher, the editors and the reviewers. Any product that may be evaluated in this article, or claim that may be made by its manufacturer, is not guaranteed or endorsed by the publisher.
