# Supplementary material for: Pan-neutralizing, germline-encoded antibodies against SARS-CoV-2: Addressing the long-term problem of escape variants
Source: Front Immunol. 2022 Oct 28;13:1032574. doi: 10.3389/fimmu.2022.1032574 (PMC9650492; doi:10.3389/fimmu.2022.1032574)
Supplement: Supplementary file 1 [file DataSheet_1.pdf]

Supplementary Materials and Methods

PRNT assay:

Serum samples with pre-determined plaque reduction neutralization test (PRNT) results were provided by Kathleen McDonough, PhD, at Wadsworth Center, NYSDOH. Briefly, the PRNT procedure was carried out by mixing 200 plaque forming units (PFU) of live SARS-CoV-2 virus in 100 µL with 100 µL of 2-fold serial dilutions of the serum samples. After incubation at 37°C for 1 hour, the virus/serum mixture was equally divided and used to infect 2 wells of Vero E6 cells (100 µL each ≈100 input PFU each). At three days post infection, the plates were stained, and the number of the plaques on the plates was counted 1 day after staining. The $PRNT_{90}$ test result was recorded as the highest serum dilution that results in over 90% reduction in the number of plaques.

NeutAb detection by NeutraXpress™:

The NeutraXpress™ test was performed on all serum samples as follows. The tests and the diluent included in the kit were brought from 4°C storage to room temperature and allowed to sit for 30 min. Frozen serum samples were thawed, mixed well and spun at top speed in a microcentrifuge for 2 minutes. 15 µL serum sample was premixed with 15 µL of the diluent, and the mixture was loaded onto the sample well #2. After incubation at room temp for one minute, two drops of the diluent were added to the sample well #2, and three drops of diluent to the control well #1. The results were recorded after 15, 30, and 60 minutes of incubation at room temperature.

Respiratory Infection Panel:

To further assess the specificity and cross-reactivity of the NeutraXpress™ test kit, a panel of 12 non-COVID-19 respiratory infection serum samples (Respiratory Infection Panel, code CF 8037) were obtained from Aalto Bio Reagents (https://www.aaltobioreagents.com/). The serological data of these samples are summarized in the Table below.

	Sample Description										
Sample ID	1	2	3	4	5	6	7	8	9	10	11
RP001	(-) MERS	RSV IgG	Flu A IgM	Flu B IgG / IgM	Parainfluenza IgG		Enterovirus IgM	Myco IgG		B. pertussis IgM	C. pneumoniae IgG
RP003	(-) MERS	RSV IgG	Flu A IgM	Flu B IgM	Parainfluenza IgG	Adenovirus IgG		Myco IgG			C. pneumoniae IgG
RP009	(-) MERS	RSV IgG	Flu A IgM	Flu B IgG / IgM	Parainfluenza IgG			Myco IgG		B. pertussis IgM	C. pneumoniae IgG
RP019	(-) MERS	RSV IgG	Flu A IgM	Flu B IgG	Parainfluenza IgG		Enterovirus IgG	Myco IgG / IgM	Legionella (+)	B. pertussis IgM	
RP027	(-) MERS	RSV IgG	Flu A IgM	Flu B IgG	Parainfluenza IgG			Myco IgG / IgM			C. pneumoniae IgG
RP030	(-) MERS	RSV IgG	Flu A IgM / IgA / IgG	Flu B IgA / IgM	Parainfluenza IgG	Adenovirus IgG		Myco IgG		B. pertussis IgM	
RP031	(-) MERS	RSV IgG	Flu A IgM	Flu B IgG / IgM	Parainfluenza IgG	Adenovirus IgG	Enterovirus IgG	Myco IgG / IgM	Legionella (+)	B. pertussis IgM	C. pneumoniae IgG
RP042	MERS Eqv	RSV IgG	Flu A IgM	Flu B IgG / IgM	Parainfluenza IgG	Adenovirus IgG	Enterovirus IgG	Myco IgG / IgM		B. pertussis IgM	
RP060	(-) MERS	RSV IgG	Flu A IgM	Flu B IgG	Parainfluenza IgG		Enterovirus IgG	Myco IgG			C. pneumoniae IgG
RP063	(-) MERS	RSV IgG	Flu A IgM	Flu B IgG / IgM	Parainfluenza IgG	Adenovirus IgG		Myco IgG	Legionella (+)		C. pneumoniae IgM
RP082	(-) MERS	RSV IgG	Flu A IgG / IgM / IgA	Flu B IgG / IgA / IgM	Parainfluenza IgG	Adenovirus IgG					C. pneumoniae IgG
RP095	(-) MERS	RSV IgG	Flu A IgM / IgA	Flu B IgG / IgA / IgM	Parainfluenza IgG		Enterovirus IgG	Myco IgG / IgM		B. pertussis IgM	C. pneumoniae IgG

All samples were collected at US FDA-licensed blood centers. Donors called the center within 3 days post-collection to report fevers and additional symptoms. All samples were negative for HIV, HBV, HCV, and syphilis. Pre-covid collection dates (11/2015 – 9/2019). Extensive clinical data (age, sex, location, fever duration, cough, rash, headache, travel etc.) are on file.
