# Supplementary material for: Pan-neutralizing, germline-encoded antibodies against SARS-CoV-2: Addressing the long-term problem of escape variants
Source: Front Immunol. 2022 Oct 28;13:1032574. doi: 10.3389/fimmu.2022.1032574 (PMC9650492; doi:10.3389/fimmu.2022.1032574)
Supplement: Supplementary file 2 [file DataSheet_2.pdf]

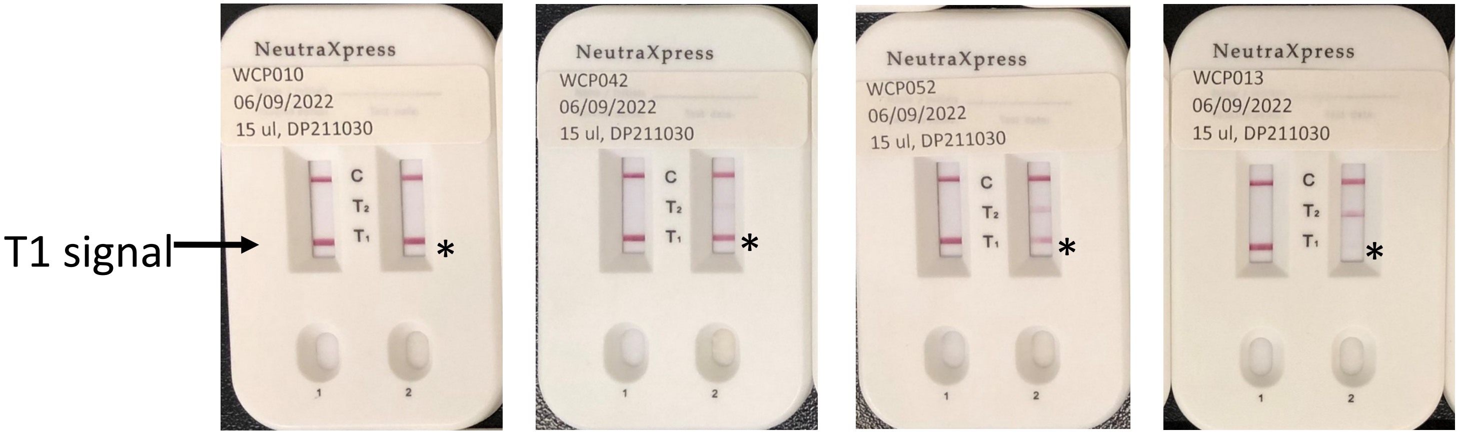

T1 inhibition:	no	borderline	clear	complete
Recorded as:	(-)	(+/-)	(+)	(++)

Supplementary Figure 1. NeutraXpress™ test results are recorded in four categories. Serum samples were added in sample well #2, whereas diluent was added in sample well #1 as control. The reduction of T1 intensity (*) in the sample well compared to that in the diluent well indicates the presence of neutAbs.

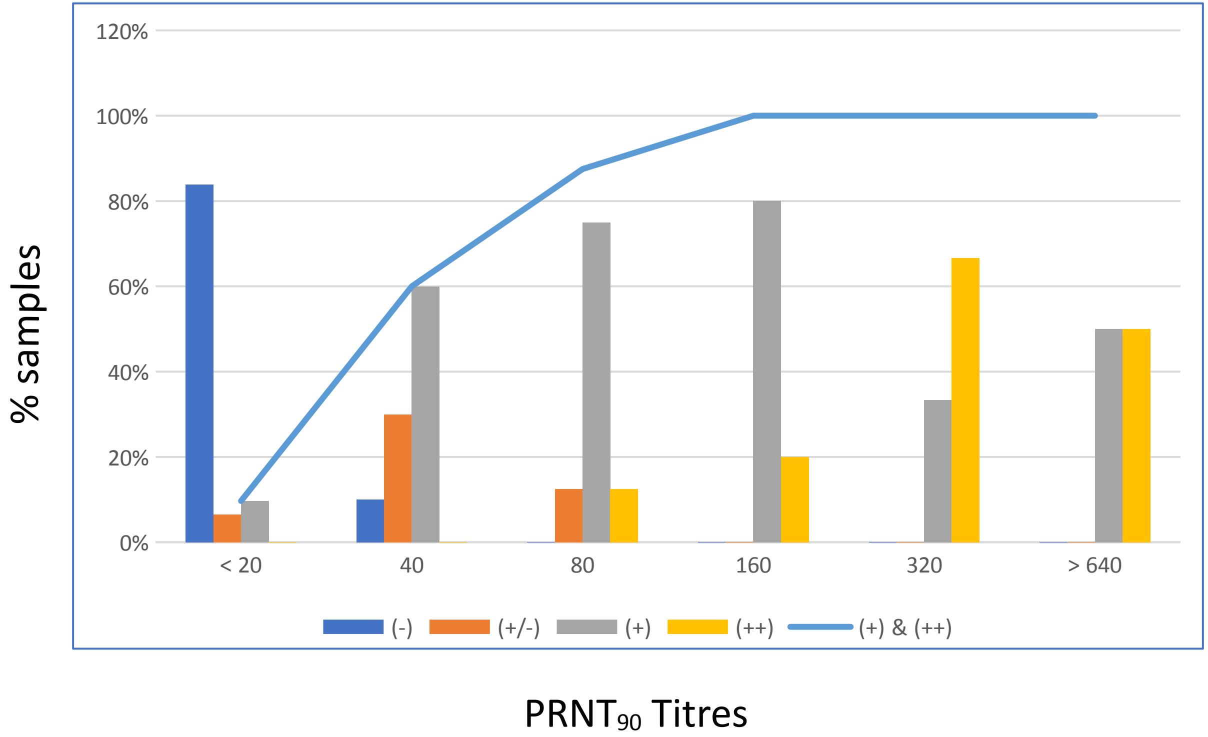

Supplementary Figure 2. NeutraXpress™ test results correlate well with the $PRNT_{90}$ titres of serum samples from COVID-19 patients. The sensitivity of detecting $PRNT_{90}$ titres >80 is 88% when (+) and (++) are combined.

Antagen DISCOVID™ IgM/IgG Test Kit

Time from onset of symptoms to blood collection (days)	Number of PCR+ Samples	PCR and LFIA agreement	LFIA IgM+ only	LFIA IgG+ only	LFIA IgM+ and IgG+	LFIA IgM+ and/or IgG+	LFIA IgM a[illegible] IgG negati[illegible]
<7	17	4/17 (24%)	2	1	1	4	13
7-10	12	12/12 (100%)	2	0	10	12	0
11-14	9	9/9 (100%)	3	0	6	9	0
15-18	8	7/8 (88%)	1	1	5	7	1
19-21	3	3/3 (100%)	0	0	3	3	0
>21	34	34/34 (100%)	0	7	27	34	0
Asymptomatic	8	5/8 (63%)	2	2	1	5	3
Total	91	74/91 (81%)	10	11	53	74	17

Samples (December 2019)	SARS-CoV-2 RT PCR result	LFIA IgM+ only	LFIA IgG + only	LFIA IgM+and IgG+	LFIA IgM+ and/or IgG+	LFIA IgM and IgG negative	Negative control Agreement
119	Not Performed	3	1	1	5	114	114/119 (96%)

Supplementary Table 1. Detection of RBD-specific IgM and/or IgG in COVID-19 patients and historial controls with Antagen's DISCOVID™ rapid tests. The samples contain (i) sera from 91 patients hospitalized in Beth Israel Deaconess Medical Center for COVID-19 or suspected COVID-19; (ii) sera from 119 patients, obtained/collected in BIDMC HLA lab prior to the onset of SARS-CoV-2 pandemic in MA before December 2019.

		Comparator assay (RT-PCR and “historical negative”)	
		**Positive**	**Negative**
**Candidate assay Antagen DISCOVID™ COVID-19 IgM/IgG Antibody Rapid Test**	Reactive for either IgM+ or IgG+ or both	51	5
	Equivocal	0	0
	Non-Reactive	4	141
	**Total**	**55**	**146**

PPA = 92.7% (95% CI of 82.7-97.1%) (Wilson Score Method)
NPA = 96.6% (95% CI of 92.2-98.5%) (Wilson Score Method)

Supplementary Table 2. Sensitiviy and specificity of Antagen’s DISCOVID™ rapid tests to detect COVID-19. Only patients with symptom onset ≥ 7 days were included in the correlation testing with the comparator assay.

T1 inhibition	PRNT90	< 20	40	80	160	320	> 640
no	(-)	26 (84%)	1 (10%)	0 (0%)	0 (0%)	0 (0%)	0 (0%)
borderline	(+/-)	2 (6%)	3 (30%)	1 (13%)	0 (0%)	0 (0%)	0 (0%)
clear	(+)	3 (10%)	6 (60%)	6 (75%)	4 (80%)	3 (33%)	2 (50%)
complete	(++)	0 (0%)	0 (0%)	1 (13%)	1 (20%)	6 (67%)	2 (50%)
	(+) & (++)	3 (10%)	6 (60%)	7 (88%)	5 (100%)	9 (100%)	4 (100%)

Supplementary Table 3. NeutraXpress™ test results correlate well with the $PRNT_{90}$ titres of serum samples from COVID-19 patients. Percentages of the total patients in the specified category are shown in the parentheses. The sensitivity of detecting $PRNT_{90}$ titres >80 is 88% when (+) and (++) are combined.

	Sample Description											
Sample ID	1	2	3	4	5	6	7	8	9	10	11	NeutraXpress test result
RP001	(-) MERS	RSV IgG	Flu A IgM	Flu B IgG / IgM	Parainfluenza IgG		Enterovirus IgM	Myco IgG		B. pertussis IgM	C. pneumoniae IgG	(-)
RP003	(-) MERS	RSV IgG	Flu A IgM	Flu B IgM	Parainfluenza IgG	Adenovirus IgG		Myco IgG			C. pneumoniae IgG	(-)
RP009	(-) MERS	RSV IgG	Flu A IgM	Flu B IgG / IgM	Parainfluenza IgG			Myco IgG		B. pertussis IgM	C. pneumoniae IgG	(-)
RP019	(-) MERS	RSV IgG	Flu A IgM	Flu B IgG	Parainfluenza IgG		Enterovirus IgG	Myco IgG / IgM	Legionella (+)	B. pertussis IgM		(-)
RP027	(-) MERS	RSV IgG	Flu A IgM	Flu B IgG	Parainfluenza IgG			Myco IgG / IgM			C. pneumoniae IgG	(-)
RP030	(-) MERS	RSV IgG	Flu A IgM / IgA / IgG	Flu B IgA / IgM	Parainfluenza IgG	Adenovirus IgG		Myco IgG		B. pertussis IgM		(-)
RP031	(-) MERS	RSV IgG	Flu A IgM	Flu B IgG / IgM	Parainfluenza IgG	Adenovirus IgG	Enterovirus IgG	Myco IgG / IgM	Legionella (+)	B. pertussis IgM	C. pneumoniae IgG	(-)
RP042	MERS Eqv	RSV IgG	Flu A IgM	Flu B IgG / IgM	Parainfluenza IgG	Adenovirus IgG	Enterovirus IgG	Myco IgG / IgM		B. pertussis IgM		(-)
RP060	(-) MERS	RSV IgG	Flu A IgM	Flu B IgG	Parainfluenza IgG		Enterovirus IgG	Myco IgG			C. pneumoniae IgG	(-)
RP063	(-) MERS	RSV IgG	Flu A IgM	Flu B IgG / IgM	Parainfluenza IgG	Adenovirus IgG		Myco IgG	Legionella (+)		C. pneumoniae IgM	(-)
RP082	(-) MERS	RSV IgG	Flu A IgG / IgM / IgA	Flu B IgG / IgA / IgM	Parainfluenza IgG	Adenovirus IgG					C. pneumoniae IgG	(-)
RP095	(-) MERS	RSV IgG	Flu A IgM / IgA	Flu B IgG / IgA / IgM	Parainfluenza IgG		Enterovirus IgG	Myco IgG / IgM		B. pertussis IgM	C. pneumoniae IgG	(-)

Supplementary Table 4. No cross reactivity of NeutraXpress™ test was detected from a panel of non-COVID-19 respiratory infection serum samples. NeutraXpress™ test also showed complete negative results in 75 historical samples collected in BIDMC HLA lab prior to the onset of SARS-CoV-2 pandemic in MA before December 2019 (not shown).
